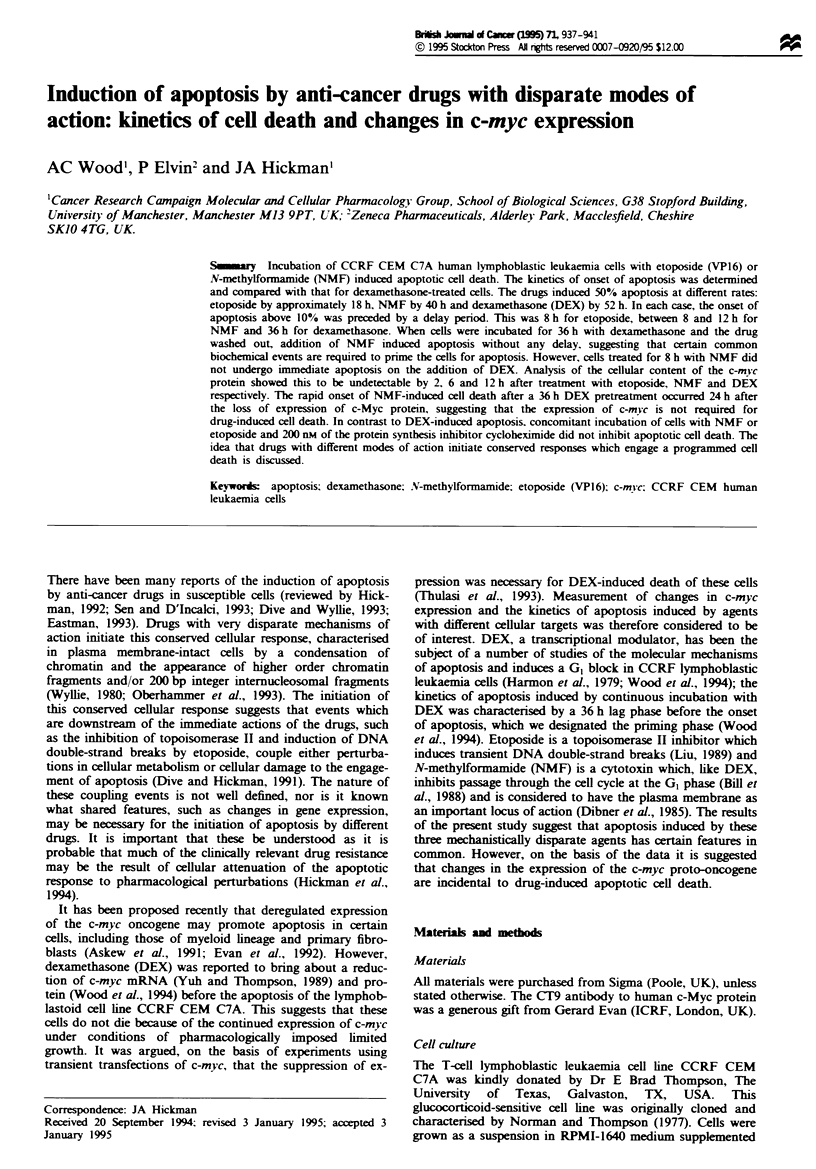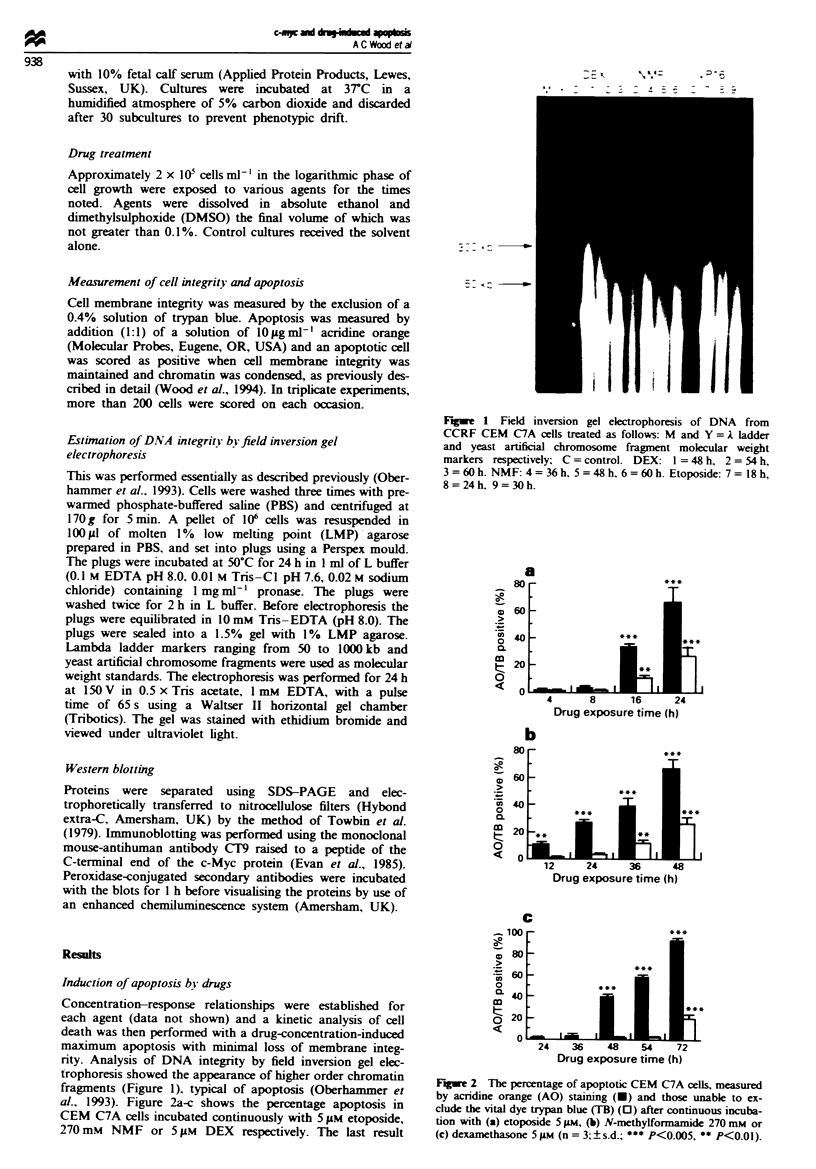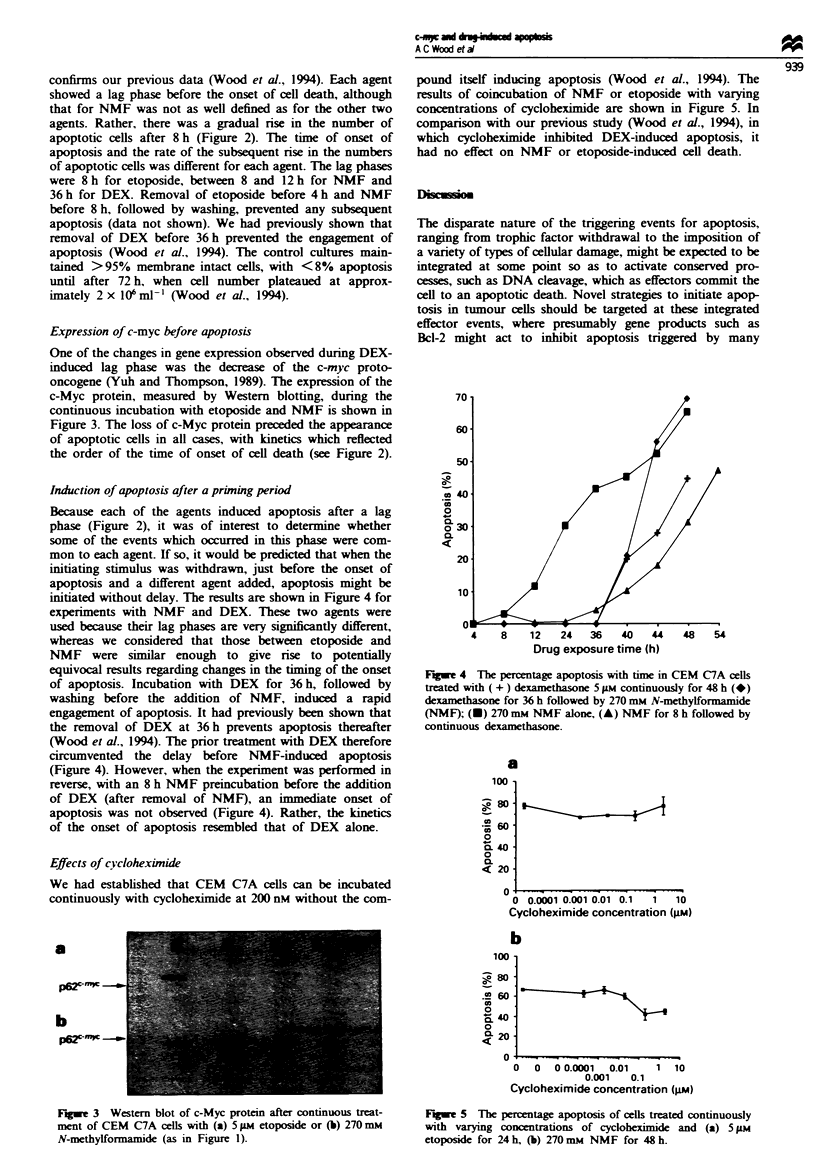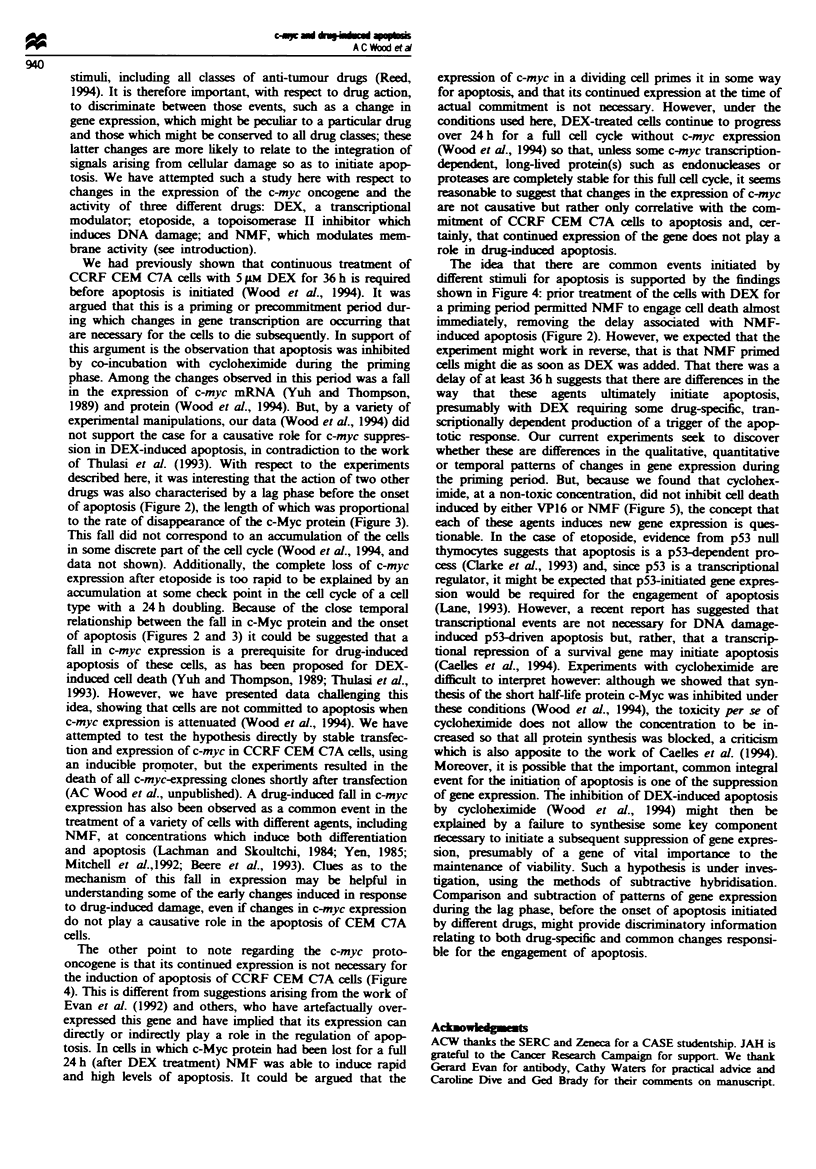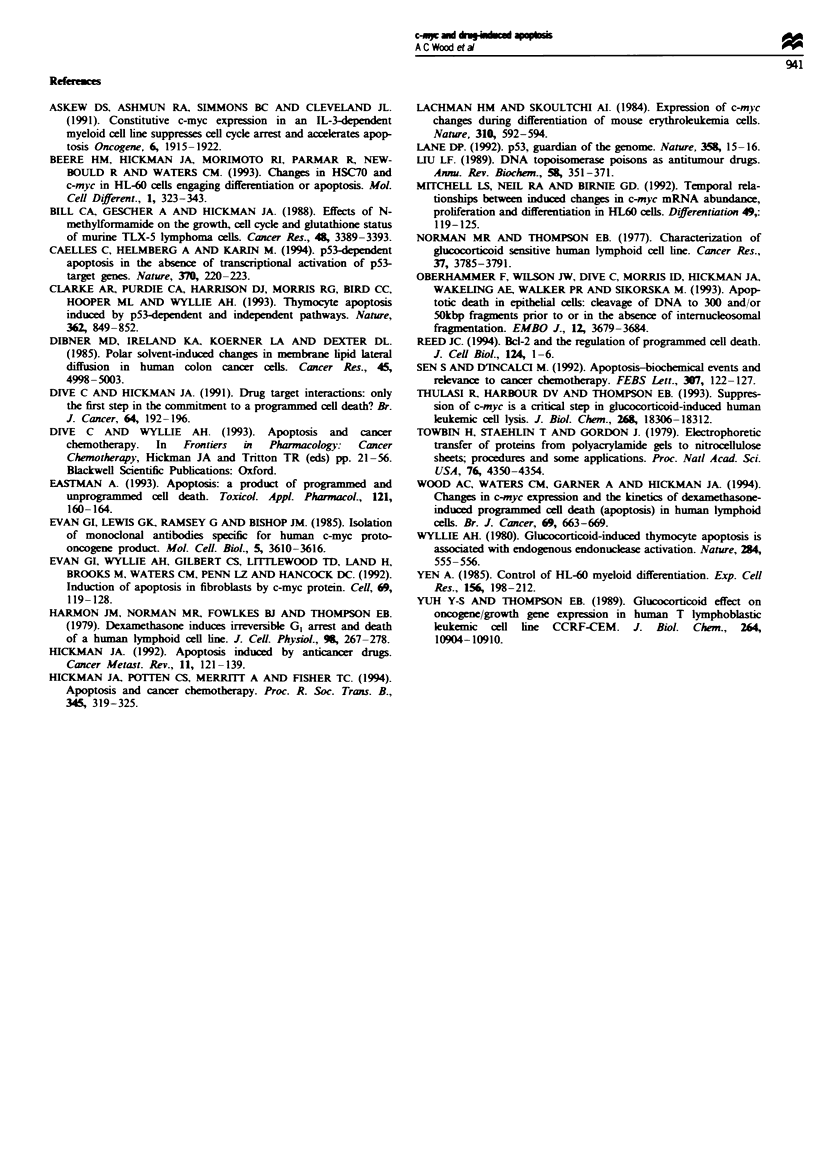# Induction of apoptosis by anti-cancer drugs with disparate modes of action: kinetics of cell death and changes in c-myc expression.

**DOI:** 10.1038/bjc.1995.181

**Published:** 1995-05

**Authors:** A. C. Wood, P. Elvin, J. A. Hickman

**Affiliations:** Cancer Research Campaign Molecular and Cellular Pharmacology Group, School of Biological Sciences, University of Manchester, UK.

## Abstract

**Images:**


British Journal of Cancer (1995) 71, 937-941

© 1995 Stockton Press All rights reserved 0007-0920/95 $12.00

### Induction of apoptosis by anti-cancer drugs with disparate modes of action: kinetics of cell death and changes in c-*myc* expression

AC Wood[1], P Elvin[2] and JA Hickman[1]

[1]*Cancer Research Campaign Molecular and Cellular Pharmacology Group, School of Biological Sciences, G38 Stopford Building, University of Manchester, Manchester M13 9PT, UK;* [2]*Zeneca Pharmaceuticals, Alderley Park, Macclesfield, Cheshire SK10 4TG, UK.*

**Summary** Incubation of CCRF CEM C7A human lymphoblastic leukaemia cells with etoposide (VP16) or *N*-methylformamide (NMF) induced apoptotic cell death. The kinetics of onset of apoptosis was determined and compared with that for dexamethasone-treated cells. The drugs induced 50% apoptosis at different rates: etoposide by approximately 18 h, NMF by 40 h and dexamethasone (DEX) by 52 h. In each case, the onset of apoptosis above 10% was preceded by a delay period. This was 8 h for etoposide, between 8 and 12 h for NMF and 36 h for dexamethasone. When cells were incubated for 36 h with dexamethasone and the drug washed out, addition of NMF induced apoptosis without any delay, suggesting that certain common biochemical events are required to prime the cells for apoptosis. However, cells treated for 8 h with NMF did not undergo immediate apoptosis on the addition of DEX. Analysis of the cellular content of the c-*myc* protein showed this to be undetectable by 2, 6 and 12 h after treatment with etoposide, NMF and DEX respectively. The rapid onset of NMF-induced cell death after a 36 h DEX pretreatment occurred 24 h after the loss of expression of c-Myc protein, suggesting that the expression of c-*myc* is not required for drug-induced cell death. In contrast to DEX-induced apoptosis, concomitant incubation of cells with NMF or etoposide and 200 nM of the protein synthesis inhibitor cycloheximide did not inhibit apoptotic cell death. The idea that drugs with different modes of action initiate conserved responses which engage a programmed cell death is discussed.

**Keywords:** apoptosis; dexamethasone; *N*-methylformamide; etoposide (VP16); c-*myc*; CCRF CEM human leukaemia cells

There have been many reports of the induction of apoptosis by anti-cancer drugs in susceptible cells (reviewed by Hickman, 1992; Sen and D'Incalci, 1993; Dive and Wyllie, 1993; Eastman, 1993). Drugs with very disparate mechanisms of action initiate this conserved cellular response, characterised in plasma membrane-intact cells by a condensation of chromatin and the appearance of higher order chromatin fragments and/or 200 bp integer internucleosomal fragments (Wyllie, 1980; Oberhammer *et al.*, 1993). The initiation of this conserved cellular response suggests that events which are downstream of the immediate actions of the drugs, such as the inhibition of topoisomerase II and induction of DNA double-strand breaks by etoposide, couple either perturbations in cellular metabolism or cellular damage to the engagement of apoptosis (Dive and Hickman, 1991). The nature of these coupling events is not well defined, nor is it known what shared features, such as changes in gene expression, may be necessary for the initiation of apoptosis by different drugs. It is important that these be understood as it is probable that much of the clinically relevant drug resistance may be the result of cellular attenuation of the apoptotic response to pharmacological perturbations (Hickman *et al.*, 1994).

It has been proposed recently that deregulated expression of the c-*myc* oncogene may promote apoptosis in certain cells, including those of myeloid lineage and primary fibroblasts (Askew *et al.*, 1991; Evan *et al.*, 1992). However, dexamethasone (DEX) was reported to bring about a reduction of c-*myc* mRNA (Yuh and Thompson, 1989) and protein (Wood *et al.*, 1994) before the apoptosis of the lymphoblastoid cell line CCRF CEM C7A. This suggests that these cells do not die because of the continued expression of c-*myc* under conditions of pharmacologically imposed limited growth. It was argued, on the basis of experiments using transient transfections of c-*myc*, that the suppression of expression was necessary for DEX-induced death of these cells (Thulasi *et al.*, 1993). Measurement of changes in c-*myc* expression and the kinetics of apoptosis induced by agents with different cellular targets was therefore considered to be of interest. DEX, a transcriptional modulator, has been the subject of a number of studies of the molecular mechanisms of apoptosis and induces a $G_1$ block in CCRF lymphoblastic leukaemia cells (Harmon *et al.*, 1979; Wood *et al.*, 1994); the kinetics of apoptosis induced by continuous incubation with DEX was characterised by a 36 h lag phase before the onset of apoptosis, which we designated the priming phase (Wood *et al.*, 1994). Etoposide is a topoisomerase II inhibitor which induces transient DNA double-strand breaks (Liu, 1989) and *N*-methylformamide (NMF) is a cytotoxin which, like DEX, inhibits passage through the cell cycle at the $G_1$ phase (Bill *et al.*, 1988) and is considered to have the plasma membrane as an important locus of action (Dibner *et al.*, 1985). The results of the present study suggest that apoptosis induced by these three mechanistically disparate agents has certain features in common. However, on the basis of the data it is suggested that changes in the expression of the c-*myc* proto-oncogene are incidental to drug-induced apoptotic cell death.

### Materials and methods

### *Materials*

All materials were purchased from Sigma (Poole, UK), unless stated otherwise. The CT9 antibody to human c-Myc protein was a generous gift from Gerard Evan (ICRF, London, UK).

### *Cell culture*

The T-cell lymphoblastic leukaemia cell line CCRF CEM C7A was kindly donated by Dr E Brad Thompson, The University of Texas, Galvaston, TX, USA. This glucocorticoid-sensitive cell line was originally cloned and characterised by Norman and Thompson (1977). Cells were grown as a suspension in RPMI-1640 medium supplemented

Correspondence: JA Hickman
Received 20 September 1994; revised 3 January 1995; accepted 3 January 1995

with 10% fetal calf serum (Applied Protein Products, Lewes, Sussex, UK). Cultures were incubated at 37°C in a humidified atmosphere of 5% carbon dioxide and discarded after 30 subcultures to prevent phenotypic drift.

*Drug treatment*

Approximately $2 \times 10^5$ cells $ml^{-1}$ in the logarithmic phase of cell growth were exposed to various agents for the times noted. Agents were dissolved in absolute ethanol and dimethylsulphoxide (DMSO) the final volume of which was not greater than 0.1%. Control cultures received the solvent alone.

*Measurement of cell integrity and apoptosis*

Cell membrane integrity was measured by the exclusion of a 0.4% solution of trypan blue. Apoptosis was measured by addition (1:1) of a solution of 10 $\mu g\ ml^{-1}$ acridine orange (Molecular Probes, Eugene, OR, USA) and an apoptotic cell was scored as positive when cell membrane integrity was maintained and chromatin was condensed, as previously described in detail (Wood *et al*., 1994). In triplicate experiments, more than 200 cells were scored on each occasion.

*Estimation of DNA integrity by field inversion gel electrophoresis*

This was performed essentially as described previously (Oberhammer *et al*., 1993). Cells were washed three times with pre-warmed phosphate-buffered saline (PBS) and centrifuged at 170 *g* for 5 min. A pellet of $10^6$ cells was resuspended in 100 µl of molten 1% low melting point (LMP) agarose prepared in PBS, and set into plugs using a Perspex mould. The plugs were incubated at 50°C for 24 h in 1 ml of L buffer (0.1 M EDTA pH 8.0, 0.01 M Tris-Cl pH 7.6, 0.02 M sodium chloride) containing 1 mg $ml^{-1}$ pronase. The plugs were washed twice for 2 h in L buffer. Before electrophoresis the plugs were equilibrated in 10 mM Tris-EDTA (pH 8.0). The plugs were sealed into a 1.5% gel with 1% LMP agarose. Lambda ladder markers ranging from 50 to 1000 kb and yeast artificial chromosome fragments were used as molecular weight standards. The electrophoresis was performed for 24 h at 150 V in 0.5 × Tris acetate, 1 mM EDTA, with a pulse time of 65 s using a Waltser II horizontal gel chamber (Tribotics). The gel was stained with ethidium bromide and viewed under ultraviolet light.

*Western blotting*

Proteins were separated using SDS-PAGE and electrophoretically transferred to nitrocellulose filters (Hybond extra-C, Amersham, UK) by the method of Towbin *et al*. (1979). Immunoblotting was performed using the monoclonal mouse-antihuman antibody CT9 raised to a peptide of the C-terminal end of the c-Myc protein (Evan *et al*., 1985). Peroxidase-conjugated secondary antibodies were incubated with the blots for 1 h before visualising the proteins by use of an enhanced chemiluminescence system (Amersham, UK).

### Results

*Induction of apoptosis by drugs*

Concentration-response relationships were established for each agent (data not shown) and a kinetic analysis of cell death was then performed with a drug-concentration-induced maximum apoptosis with minimal loss of membrane integrity. Analysis of DNA integrity by field inversion gel electrophoresis showed the appearance of higher order chromatin fragments (Figure 1), typical of apoptosis (Oberhammer *et al*., 1993). Figure 2a-c shows the percentage apoptosis in CEM C7A cells incubated continuously with 5 µM etoposide, 270 mM NMF or 5 µM DEX respectively. The last result

**Figure 1** Field inversion gel electrophoresis of DNA from CCRF CEM C7A cells treated as follows: M and Y = λ ladder and yeast artificial chromosome fragment molecular weight markers respectively; C = control. DEX: 1 = 48 h, 2 = 54 h, 3 = 60 h. NMF: 4 = 36 h, 5 = 48 h, 6 = 60 h. Etoposide: 7 = 18 h, 8 = 24 h, 9 = 30 h.

**Figure 2** The percentage of apoptotic CEM C7A cells, measured by acridine orange (AO) staining (■) and those unable to exclude the vital dye trypan blue (TB) (□) after continuous incubation with (**a**) etoposide 5 µM, (**b**) *N*-methylformamide 270 mM or (**c**) dexamethasone 5 µM (n = 3; ± s.d.; *** $P < 0.005$, ** $P < 0.01$).

confirms our previous data (Wood *et al*., 1994). Each agent showed a lag phase before the onset of cell death, although that for NMF was not as well defined as for the other two agents. Rather, there was a gradual rise in the number of apoptotic cells after 8 h (Figure 2). The time of onset of apoptosis and the rate of the subsequent rise in the numbers of apoptotic cells was different for each agent. The lag phases were 8 h for etoposide, between 8 and 12 h for NMF and 36 h for DEX. Removal of etoposide before 4 h and NMF before 8 h, followed by washing, prevented any subsequent apoptosis (data not shown). We had previously shown that removal of DEX before 36 h prevented the engagement of apoptosis (Wood *et al*., 1994). The control cultures maintained >95% membrane intact cells, with <8% apoptosis until after 72 h, when cell number plateaued at approximately $2 \times 10^6\,ml^{-1}$ (Wood *et al*., 1994).

### *Expression of c-*myc *before apoptosis*

One of the changes in gene expression observed during DEX-induced lag phase was the decrease of the c-*myc* proto-oncogene (Yuh and Thompson, 1989). The expression of the c-Myc protein, measured by Western blotting, during the continuous incubation with etoposide and NMF is shown in Figure 3. The loss of c-Myc protein preceded the appearance of apoptotic cells in all cases, with kinetics which reflected the order of the time of onset of cell death (see Figure 2).

### *Induction of apoptosis after a priming period*

Because each of the agents induced apoptosis after a lag phase (Figure 2), it was of interest to determine whether some of the events which occurred in this phase were common to each agent. If so, it would be predicted that when the initiating stimulus was withdrawn, just before the onset of apoptosis and a different agent added, apoptosis might be initiated without delay. The results are shown in Figure 4 for experiments with NMF and DEX. These two agents were used because their lag phases are very significantly different, whereas we considered that those between etoposide and NMF were similar enough to give rise to potentially equivocal results regarding changes in the timing of the onset of apoptosis. Incubation with DEX for 36 h, followed by washing before the addition of NMF, induced a rapid engagement of apoptosis. It had previously been shown that the removal of DEX at 36 h prevents apoptosis thereafter (Wood *et al*., 1994). The prior treatment with DEX therefore circumvented the delay before NMF-induced apoptosis (Figure 4). However, when the experiment was performed in reverse, with an 8 h NMF preincubation before the addition of DEX (after removal of NMF), an immediate onset of apoptosis was not observed (Figure 4). Rather, the kinetics of the onset of apoptosis resembled that of DEX alone.

### *Effects of cycloheximide*

We had established that CEM C7A cells can be incubated continuously with cycloheximide at 200 nM without the compound itself inducing apoptosis (Wood *et al*., 1994). The results of coincubation of NMF or etoposide with varying concentrations of cycloheximide are shown in Figure 5. In comparison with our previous study (Wood *et al*., 1994), in which cycloheximide inhibited DEX-induced apoptosis, it had no effect on NMF or etoposide-induced cell death.

### Discussion

The disparate nature of the triggering events for apoptosis, ranging from trophic factor withdrawal to the imposition of a variety of types of cellular damage, might be expected to be integrated at some point so as to activate conserved processes, such as DNA cleavage, which as effectors commit the cell to an apoptotic death. Novel strategies to initiate apoptosis in tumour cells should be targeted at these integrated effector events, where presumably gene products such as Bcl-2 might act to inhibit apoptosis triggered by many

**Figure 3** Western blot of c-Myc protein after continuous treatment of CEM C7A cells with (**a**) 5 μM etoposide or (**b**) 270 mM *N*-methylformamide (as in Figure 1).

**Figure 4** The percentage apoptosis with time in CEM C7A cells treated with ( + ) dexamethasone 5 μM continuously for 48 h (◆) dexamethasone for 36 h followed by 270 mM *N*-methylformamide (NMF); (■) 270 mM NMF alone, (▲) NMF for 8 h followed by continuous dexamethasone.

**Figure 5** The percentage apoptosis of cells treated continuously with varying concentrations of cycloheximide and (**a**) 5 μM etoposide for 24 h, (**b**) 270 mM NMF for 48 h.

stimuli, including all classes of anti-tumour drugs (Reed, 1994). It is therefore important, with respect to drug action, to discriminate between those events, such as a change in gene expression, which might be peculiar to a particular drug and those which might be conserved to all drug classes; these latter changes are more likely to relate to the integration of signals arising from cellular damage so as to initiate apoptosis. We have attempted such a study here with respect to changes in the expression of the c-*myc* oncogene and the activity of three different drugs: DEX, a transcriptional modulator; etoposide, a topoisomerase II inhibitor which induces DNA damage; and NMF, which modulates membrane activity (see introduction).

We had previously shown that continuous treatment of CCRF CEM C7A cells with 5 $\mu$M DEX for 36 h is required before apoptosis is initiated (Wood *et al*., 1994). It was argued that this is a priming or precommitment period during which changes in gene transcription are occurring that are necessary for the cells to die subsequently. In support of this argument is the observation that apoptosis was inhibited by co-incubation with cycloheximide during the priming phase. Among the changes observed in this period was a fall in the expression of c-*myc* mRNA (Yuh and Thompson, 1989) and protein (Wood *et al*., 1994). But, by a variety of experimental manipulations, our data (Wood *et al*., 1994) did not support the case for a causative role for c-*myc* suppression in DEX-induced apoptosis, in contradiction to the work of Thulasi *et al*. (1993). With respect to the experiments described here, it was interesting that the action of two other drugs was also characterised by a lag phase before the onset of apoptosis (Figure 2), the length of which was proportional to the rate of disappearance of the c-Myc protein (Figure 3). This fall did not correspond to an accumulation of the cells in some discrete part of the cell cycle (Wood *et al*., 1994, and data not shown). Additionally, the complete loss of c-*myc* expression after etoposide is too rapid to be explained by an accumulation at some check point in the cell cycle of a cell type with a 24 h doubling. Because of the close temporal relationship between the fall in c-Myc protein and the onset of apoptosis (Figures 2 and 3) it could be suggested that a fall in c-*myc* expression is a prerequisite for drug-induced apoptosis of these cells, as has been proposed for DEX-induced cell death (Yuh and Thompson, 1989; Thulasi *et al*., 1993). However, we have presented data challenging this idea, showing that cells are not committed to apoptosis when c-*myc* expression is attenuated (Wood *et al*., 1994). We have attempted to test the hypothesis directly by stable transfection and expression of c-*myc* in CCRF CEM C7A cells, using an inducible promoter, but the experiments resulted in the death of all c-*myc*-expressing clones shortly after transfection (AC Wood *et al*., unpublished). A drug-induced fall in c-*myc* expression has also been observed as a common event in the treatment of a variety of cells with different agents, including NMF, at concentrations which induce both differentiation and apoptosis (Lachman and Skoultchi, 1984; Yen, 1985; Mitchell *et al*.,1992; Beere *et al*., 1993). Clues as to the mechanism of this fall in expression may be helpful in understanding some of the early changes induced in response to drug-induced damage, even if changes in c-*myc* expression do not play a causative role in the apoptosis of CEM C7A cells.

The other point to note regarding the c-*myc* proto-oncogene is that its continued expression is not necessary for the induction of apoptosis of CCRF CEM C7A cells (Figure 4). This is different from suggestions arising from the work of Evan *et al*. (1992) and others, who have artefactually over-expressed this gene and have implied that its expression can directly or indirectly play a role in the regulation of apoptosis. In cells in which c-Myc protein had been lost for a full 24 h (after DEX treatment) NMF was able to induce rapid and high levels of apoptosis. It could be argued that the expression of c-*myc* in a dividing cell primes it in some way for apoptosis, and that its continued expression at the time of actual commitment is not necessary. However, under the conditions used here, DEX-treated cells continue to progress over 24 h for a full cell cycle without c-*myc* expression (Wood *et al*., 1994) so that, unless some c-*myc* transcription-dependent, long-lived protein(s) such as endonucleases or proteases are completely stable for this full cell cycle, it seems reasonable to suggest that changes in the expression of c-*myc* are not causative but rather only correlative with the commitment of CCRF CEM C7A cells to apoptosis and, certainly, that continued expression of the gene does not play a role in drug-induced apoptosis.

The idea that there are common events initiated by different stimuli for apoptosis is supported by the findings shown in Figure 4: prior treatment of the cells with DEX for a priming period permitted NMF to engage cell death almost immediately, removing the delay associated with NMF-induced apoptosis (Figure 2). However, we expected that the experiment might work in reverse, that is that NMF primed cells might die as soon as DEX was added. That there was a delay of at least 36 h suggests that there are differences in the way that these agents ultimately initiate apoptosis, presumably with DEX requiring some drug-specific, transcriptionally dependent production of a trigger of the apoptotic response. Our current experiments seek to discover whether these are differences in the qualitative, quantitative or temporal patterns of changes in gene expression during the priming period. But, because we found that cycloheximide, at a non-toxic concentration, did not inhibit cell death induced by either VP16 or NMF (Figure 5), the concept that each of these agents induces new gene expression is questionable. In the case of etoposide, evidence from p53 null thymocytes suggests that apoptosis is a p53-dependent process (Clarke *et al*., 1993) and, since p53 is a transcriptional regulator, it might be expected that p53-initiated gene expression would be required for the engagement of apoptosis (Lane, 1993). However, a recent report has suggested that transcriptional events are not necessary for DNA damage-induced p53-driven apoptosis but, rather, that a transcriptional repression of a survival gene may initiate apoptosis (Caelles *et al*., 1994). Experiments with cycloheximide are difficult to interpret however: although we showed that synthesis of the short half-life protein c-Myc was inhibited under these conditions (Wood *et al*., 1994), the toxicity *per se* of cycloheximide does not allow the concentration to be increased so that all protein synthesis was blocked, a criticism which is also apposite to the work of Caelles *et al*. (1994). Moreover, it is possible that the important, common integral event for the initiation of apoptosis is one of the suppression of gene expression. The inhibition of DEX-induced apoptosis by cycloheximide (Wood *et al*., 1994) might then be explained by a failure to synthesise some key component necessary to initiate a subsequent suppression of gene expression, presumably of a gene of vital importance to the maintenance of viability. Such a hypothesis is under investigation, using the methods of subtractive hybridisation. Comparison and subtraction of patterns of gene expression during the lag phase, before the onset of apoptosis initiated by different drugs, might provide discriminatory information relating to both drug-specific and common changes responsible for the engagement of apoptosis.

**Acknowledgments**

ACW thanks the SERC and Zeneca for a CASE studentship. JAH is grateful to the Cancer Research Campaign for support. We thank Gerard Evan for antibody, Cathy Waters for practical advice and Caroline Dive and Ged Brady for their comments on manuscript.

### References

ASKEW DS, ASHMUN RA, SIMMONS BC AND CLEVELAND JL. (1991). Constitutive c-myc expression in an IL-3-dependent myeloid cell line suppresses cell cycle arrest and accelerates apoptosis *Oncogene*, **6**, 1915–1922.

BEERE HM, HICKMAN JA, MORIMOTO RI, PARMAR R, NEWBOULD R AND WATERS CM. (1993). Changes in HSC70 and c-*myc* in HL-60 cells engaging differentiation or apoptosis. *Mol. Cell Different.*, **1**, 323–343.

BILL CA, GESCHER A AND HICKMAN JA. (1988). Effects of N-methylformamide on the growth, cell cycle and glutathione status of murine TLX-5 lymphoma cells. *Cancer Res.*, **48**, 3389–3393.

CAELLES C, HELMBERG A AND KARIN M. (1994). p53-dependent apoptosis in the absence of transcriptional activation of p53-target genes. *Nature*, **370**, 220–223.

CLARKE AR, PURDIE CA, HARRISON DJ, MORRIS RG, BIRD CC, HOOPER ML AND WYLLIE AH. (1993). Thymocyte apoptosis induced by p53-dependent and independent pathways. *Nature*, **362**, 849–852.

DIBNER MD, IRELAND KA, KOERNER LA AND DEXTER DL. (1985). Polar solvent-induced changes in membrane lipid lateral diffusion in human colon cancer cells. *Cancer Res.*, **45**, 4998–5003.

DIVE C AND HICKMAN JA. (1991). Drug target interactions: only the first step in the commitment to a programmed cell death? *Br. J. Cancer*, **64**, 192–196.

DIVE C AND WYLLIE AH. (1993). Apoptosis and cancer chemotherapy. In *Frontiers in Pharmacology: Cancer Chemotherapy*, Hickman JA and Tritton TR (eds) pp. 21–56. Blackwell Scientific Publications: Oxford.

EASTMAN A. (1993). Apoptosis: a product of programmed and unprogrammed cell death. *Toxicol. Appl. Pharmacol.*, **121**, 160–164.

EVAN GI, LEWIS GK, RAMSEY G AND BISHOP JM. (1985). Isolation of monoclonal antibodies specific for human c-myc proto-oncogene product. *Mol. Cell. Biol.*, **5**, 3610–3616.

EVAN GI, WYLLIE AH, GILBERT CS, LITTLEWOOD TD, LAND H, BROOKS M, WATERS CM, PENN LZ AND HANCOCK DC. (1992). Induction of apoptosis in fibroblasts by c-myc protein. *Cell*, **69**, 119–128.

HARMON JM, NORMAN MR, FOWLKES BJ AND THOMPSON EB. (1979). Dexamethasone induces irreversible $G_1$ arrest and death of a human lymphoid cell line. *J. Cell. Physiol.*, **98**, 267–278.

HICKMAN JA. (1992). Apoptosis induced by anticancer drugs. *Cancer Metast. Rev.*, **11**, 121–139.

HICKMAN JA, POTTEN CS, MERRITT A AND FISHER TC. (1994). Apoptosis and cancer chemotherapy. *Proc. R. Soc. Trans. B.*, **345**, 319–325.

LACHMAN HM AND SKOULTCHI AI. (1984). Expression of c-*myc* changes during differentiation of mouse erythroleukemia cells. *Nature*, **310**, 592–594.

LANE DP. (1992). p53, guardian of the genome. *Nature*, **358**, 15–16.

LIU LF. (1989). DNA topoisomerase poisons as antitumour drugs. *Annu. Rev. Biochem.*, **58**, 351–371.

MITCHELL LS, NEIL RA AND BIRNIE GD. (1992). Temporal relationships between induced changes in c-*myc* mRNA abundance, proliferation and differentiation in HL60 cells. *Differentiation* **49**,: 119–125.

NORMAN MR AND THOMPSON EB. (1977). Characterization of glucocorticoid sensitive human lymphoid cell line. *Cancer Res.*, **37**, 3785–3791.

OBERHAMMER F, WILSON JW, DIVE C, MORRIS ID, HICKMAN JA, WAKELING AE, WALKER PR AND SIKORSKA M. (1993). Apoptotic death in epithelial cells: cleavage of DNA to 300 and/or 50kbp fragments prior to or in the absence of internucleosomal fragmentation. *EMBO J.*, **12**, 3679–3684.

REED JC. (1994). Bcl-2 and the regulation of programmed cell death. *J. Cell Biol.*, **124**, 1–6.

SEN S AND D'INCALCI M. (1992). Apoptosis–biochemical events and relevance to cancer chemotherapy. *FEBS Lett.*, **307**, 122–127.

THULASI R, HARBOUR DV AND THOMPSON EB. (1993). Suppression of c-*myc* is a critical step in glucocorticoid-induced human leukemic cell lysis. *J. Biol. Chem.*, **268**, 18306–18312.

TOWBIN H, STAEHLIN T AND GORDON J. (1979). Electrophoretic transfer of proteins from polyacrylamide gels to nitrocellulose sheets; procedures and some applications. *Proc. Natl Acad. Sci. USA*, **76**, 4350–4354.

WOOD AC, WATERS CM, GARNER A AND HICKMAN JA. (1994). Changes in c-*myc* expression and the kinetics of dexamethasone-induced programmed cell death (apoptosis) in human lymphoid cells. *Br. J. Cancer*, **69**, 663–669.

WYLLIE AH. (1980). Glucocorticoid-induced thymocyte apoptosis is associated with endogenous endonuclease activation. *Nature*, **284**, 555–556.

YEN A. (1985). Control of HL-60 myeloid differentiation. *Exp. Cell Res.*, **156**, 198–212.

YUH Y-S AND THOMPSON EB. (1989). Glucocorticoid effect on oncogene/growth gene expression in human T lymphoblastic leukemic cell line CCRF-CEM. *J. Biol. Chem.*, **264**, 10904–10910.